# Soluble ST2 is a sensitive clinical marker of ulcerative colitis evolution

**DOI:** 10.1186/s12876-016-0520-6

**Published:** 2016-08-26

**Authors:** David Díaz-Jiménez, Marjorie De la Fuente, Karen Dubois-Camacho, Glauben Landskron, Janitza Fuentes, Tamara Pérez, María Julieta González, Daniela Simian, Marcela A. Hermoso, Rodrigo Quera

**Affiliations:** 1 Programa Disciplinario de Inmunología, Instituto de Ciencias Biomédicas, Facultad de Medicina, Universidad de Chile, Santiago, CL 8380453 Chile; 2 Subdirección de Investigación, Dirección Académica, Clínica Las Condes, Santiago, CL 7591018 Chile; 3 Unidad de Hígado y Gastroenterología, Instituto Chileno-Japonés de Enfermedades Digestivas, Hospital San Borja-Arriarán, Santiago, CL Chile; 4 Programa disciplinario de Biología Celular, Instituto de Ciencias Biomédicas, Facultad de Medicina, Universidad de Chile, Santiago, CL 8380453 Chile; 5 Servicio de Gastroenterología, Clínica Las Condes, Santiago, CL 7591018 Chile

**Keywords:** Soluble ST2, Ulcerative colitis, Biomarker, Fecal calprotectin

## Abstract

**Background:**

The ST2/IL-33 pathway has been related to ulcerative colitis (UC), and soluble ST2 (sST2), to disease severity. We tested the potential usefulness of sST2 as a predictive marker of treatment response and patients’ outcome.

**Methods:**

Twenty-six patients with active UC were prospectively recruited and grouped according to an endoscopic score and therapy response. Colonoscopic biopsies were collected at baseline and 6 months or when patients showed clinical activity. The protocol was reinitiated in patients requiring rescue therapy. Blood and stool were collected at baseline, 1, 3, 6 and 12 months. Serum and mucosal ST2, and fecal calprotectin (FC) content were determined by ELISA and correlated to Mayo clinical and endoscopic subscore. Intestinal ST2 was evaluated by immunofluorescence. Wilcoxon signed rank test and Spearman correlations (Rs) were applied (*p* <0.05).

**Results:**

Follow-up was completed in 24 patients. sST2 levels (median and range) varied from 173.5 [136.6–274.0] to 86.5 [54.6–133.2] in responders (*p* < 0.05), and 336.3 [211.0–403.2] to 385.3 pg/mL [283.4–517.3] in non-responders at baseline and 6 months, respectively. sST2 levels correlated with Mayo clinical and endoscopic subscore, mucosal ST2 and FC (Rs = 0.57, 0.66, 0.74 and 0.42, respectively; *p* < 0.0001) and showed a trend similar to that of FC in responders. Non-responders revealed an increased ST2 content, restricted to the lamina propria’s cellular infiltrate.

**Conclusions:**

Consecutive sST2 measurement to follow changes in inflammatory activity of UC patients who respond or not to treatment identifies sST2, like FC, as a useful biomarker in predicting clinical outcome of UC patients.

**Electronic supplementary material:**

The online version of this article (doi:10.1186/s12876-016-0520-6) contains supplementary material, which is available to authorized users.

## Background

The two main subtypes of inflammatory bowel diseases (IBD), Crohn’s disease (CD) and ulcerative colitis (UC), are characterized by episodes of inflammatory activity and remission. To explain the multifactorial and polygenic nature of IBD, a scenario of chronic and uncontrolled activation of the mucosal immune response in genetically susceptible individuals, exacerbated by environmental factors, has been proposed [1].

Treatment of IBD seeks to induce and maintain remission, as well as reduce inflammation, promote mucosal healing and prevent complications [2], such that the choice of optimal therapy is crucial for patient recovery. In most cases, the disease can be controlled with conventional treatment, such as 5-aminosalicylate (5-ASA) derivatives, corticosteroids and immunosuppressants. However, patients who do not respond to these treatments eventually require biological therapy and, as a final alternative, colectomy. While clinical tools, such as assessment of symptoms/signs, performance of laboratory markers [3], colonoscopy/sigmoidoscopy and imaging modalities may allow monitoring the response to a selected therapy, only endoscopic studies definitively diagnose IBD and evaluate its activity through detection of intestinal lesions [4]; unfortunately this procedure is invasive and costly. Determination of disease activity remains challenging, with most clinical scores correlating poorly with the inflammatory state [4, 5]. Treatment of IBD patients has recently shifted from controlling symptoms to promoting endoscopic mucosal healing or deep remission [6], features that are now major endpoints in clinical trials and are highly efficient in predicting long-term remission and preventing hospitalization and surgery.

Treatment promoting mucosal healing can slow the progression of the disease [6]. In this context, laboratory biomarkers have gained importance in evaluating and predicting the response to therapy [7, 8]. Recently, key molecules that regulate mucosal immunity and IBD pathogenesis, including those in the IL-33/ST2 signaling pathway, have emerged as suitable biomarkers in inflammatory conditions such as IBD [9, 10]. In humans, *IL1RL1* expression is regulated by distal and proximal promoters that govern expression of the ST2 membrane-anchored receptor (ST2L) and a soluble isoform (sST2) generated by alternative splicing. sST2 is identical to the ST2L extracellular domain and is a decoy receptor for IL33 [11]. The IL-33/ST2 axis has been implicated in numerous other diseases, such as asthma, rheumatoid arthritis, cancer and Alzheimer’s disease [9, 12–14].

We previously proposed the sST2 variant as a potential severity biomarker, mainly in UC patients, that might allow differentiation between active and inactive disease [15]. However, sST2 behavior during the clinical course and response to therapy has not been explored. Here, we examined whether changes in serum ST2 levels can predict response to treatment, clinical activity and disease outcome when compared with other biomarkers, such as FC.

## Methods

### Patients

**Fig. 1:**
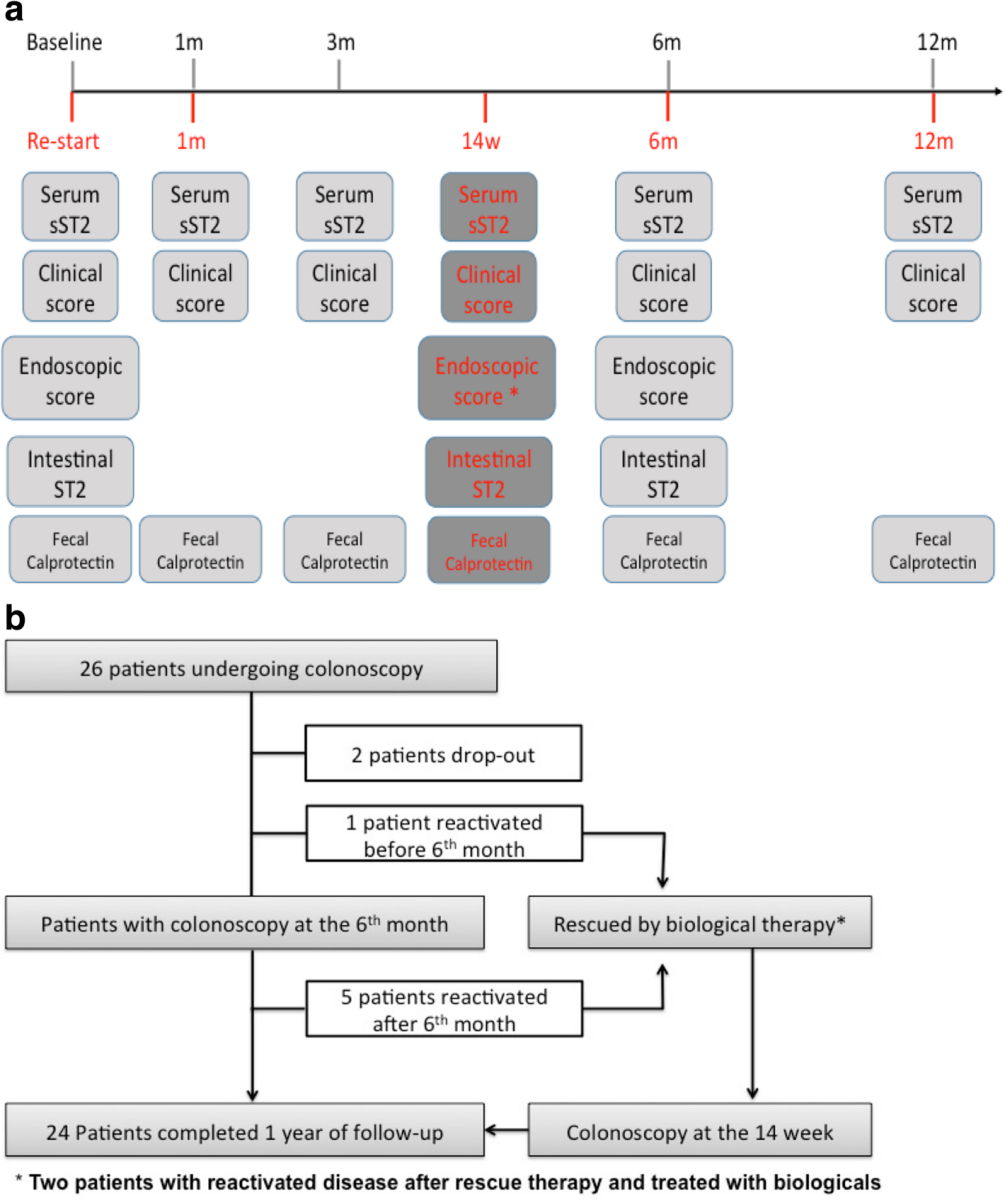
Study selection and flow-chart of UC patients’ 1-year follow-up. **a** Follow-up scheme protocol performed in active UC patients receiving conventional therapy (baseline). Responder UC patients completed the entire follow-up period. Non-responder patients re-started the follow-up with biological therapy and were examined at 1 month, 14 weeks, 6 and 12 months. Clinical subscore, FC and serum sST2 were evaluated at 1, 3, 6 and 12 months. Endoscopic subscore and intestinal ST2 were determined at baseline, 14 weeks (w) and 6 months. **b** Flow-chart of patients enrolled in the follow-up

In this observational study, we prospectively included patients with relapsed IBD from Clínica Las Condes. The diagnosis of UC was established according to international guidelines including clinical, endoscopic, histologic and radiologic criteria [16, 17]. Patients were assessed at a minimum of 3-month intervals or upon relapse. The follow-up period was 12 months in patients showing no relapse and in patients showing activity flares and requiring rescue therapy (Fig. 1a). All relapses were sufficiently severe to warrant a change in treatment.

Patients were excluded from the study based on the following criteria: incomplete ileo-colonoscopy (ileum not intubated), non-classifiable IBD or coexisting cardiopulmonary, renal, hepatic, celiac disease, neurologic, psychiatric, severe allergy and rheumatologic disease, a history of HIV, previous ileostomy or colostomy, and conditions associated with elevated FC levels [18, 19], i.e., non-steroidal anti-inflammatory or anti-coagulant drug use in the 6 months preceding enrollment, a history of erosive/ulcerative upper gastrointestinal disease within 2 months prior to the study or gastrointestinal infection within 4 weeks prior to the study and those patients treated with biological therapy. The source data was encrypted and the data extracted were anonymous. This study was performed in accordance with the Declaration of Helsinki and the protocol was approved by Clinica Las Condes Review Board of the Universidad de Chile, Chile. All patients gave written informed consent.

### Clinical activity index

During clinic visits, general well-being, stool frequency, stool consistency and presence or absence of abdominal pain, tenderness, tenesmus, rectal bleeding, and mucus in stool were recorded. Clinical activity indices were scored according to data available in patient records using the Mayo clinical subscore at baseline and at 1, 3, 6 and 12 months or, in the case of flare, during the follow-up period. Three clinical variables were graded: frequency of evacuation, amount of blood in stool, and the physician’s global assessment. A Mayo clinical subscore of 0 was defined as remission, 1–3 as mildly active disease, 4–6 as moderately active disease, and ≥7 as severely active disease [20].

All clinical decisions were made independently of ST2 protein level measurements, since detections were performed only after follow-up was completed.

### Endoscopic assessment of disease activity

Endoscopic findings were scored according to the Mayo endoscopic subscore, graded as normal (0), mild (1), moderate (2), or severe (3) disease activity. Subscore 0–1 was defined as remission and subscore ≥2 as active disease [21]. Colonoscopic biopsies were collected at baseline (colonoscopy 1) and at 6 months or if patients showed clinical signs of activity (colonoscopy 2). Patients who required rescue therapy reinitiated the protocol. Endoscopic re-evaluation in patients receiving conventional or biological therapy was conducted at 6 months or 14 weeks, respectively, or when flares were evident.

Disease activity was assessed based on the results of clinical activity scoring but blinded to quantitative FC values. Clinical response was defined as a decrease from baseline in the total Mayo score by at least three points and at least 30 %, with an accompanying decrease in the subscore for rectal bleeding of at least one point or an absolute subscore for rectal bleeding of 0 or 1. Mucosal healing was defined as an absolute subscore for endoscopy of 0 or 1. Patients who had a clinical response at each time during the follow-up were considered to have a sustained clinical response [22].

### Medical treatment

Patients enrolled in the study were treated with conventional therapy, such as 5-ASA derivatives, corticosteroids or immunomodulators (azathioprine/6-mercaptopurine), depending on disease severity. The decision to change medical treatment was based on clinical and/or endoscopic evaluation. Physicians were blinded to results of individual quantitative FC values at the time of the patient’s visit. Rescue therapy was defined as: (a) an increased dose of any previously administered medication; or (b) a change in medication to corticosteroids, immunosuppressants (azathioprine/6-mercaptopurine) or anti-TNF (infliximab/adalimumab). Information about the drug, dose and duration of treatment was obtained from the medical history.

### Measurement of ST2 and IL-33

A blood sample was collected at baseline and at 1, 3, 6 and 12 months of follow-up on the day of colonoscopy or physical examination (Fig. 1a). Serum and intestinal ST2 concentrations, as well as serum IL-33 content, were measured using an enzyme-linked immunosorbent assay (ELISA) kit for human ST2 or IL-33 (DuoSet, R&D Systems, Minneapolis, MN, USA) according to the manufacturer’s instructions and expressed as pg/mL. Blood samples were centrifuged and serum was stored at −80 °C. For ST2 and IL-33 detection, serum samples were thawed and treated with protein A/G PLUS-agarose (Santa Cruz Biotechnology, Santa Cruz, CA, USA). Protein extracts of colonic mucosa were obtained from each biopsy by homogenization using a lysis buffer supplemented with a protease inhibitor cocktail (Complete Mini, Roche Diagnostics, Basel, Switzerland) and subsequently disrupted by sonication. Levels of total intestinal ST2 were adjusted to total protein concentration determined by Bradford protein assay (Bio-Rad). The ST2 detection assay is stable over time, with a detection limit of 20 pg/mL, while the IL-33 detection assay is less stable over time, with a detection limit of 23.40 pg/mL, according to the manufacturer’s information. All samples were analyzed in duplicate; within-run and total coefficients of variation were ≤2.5 and ≤4.0 %, respectively.

Using the cut-off for sST2 previously estimated at <74.87 pg/mL [15], we determined the specificity, sensitivity, positive predictive value and negative predictive value for our patient cohort.

### Fecal calprotectin measurement

A fecal sample was collected at baseline and at, 1, 3, 6 and 12 months of follow-up (Fig. 1a) 24 h before colonoscopy or physical examination. Feces (50 mg) was resuspended in 500 μL of extraction buffer, spun for 10 min and centrifuged for 10 min at 10,000 rpm. The supernatant was immediately processed for rapid semi-quantitative test (Calprotectin 50 + 200, CerTest Biotec S.L. Spain) or stored at −80 °C for subsequent quantification using a standard ELISA with a solid bound specific antibody and biotinylated tracer antibody according to the manufacturer’s instructions (Hycult Biotech, HK325-02, The Netherlands). Detection levels were 1.6–100 ng/mL, corresponding to 40–2500 μg calprotectin/g of feces, adjusted for the dilution factor.

Clinical decisions to change therapy were based on semi-quantitative analysis of calprotectin and independent of quantitative ELISA after completed follow-up.

### Endpoints

The correlation of ST2 and FC measurements with clinical and endoscopic activity scoring was the primary endpoint of the study. Complete response (remission) was defined as the absence of symptoms, normal stool frequency, absence of rectal bleeding, general wellness based on the patient’s functional assessment score and endoscopy findings of subscore 0–1 [21]. Biological remission also implies healing of mucosa and normalization of inflammation biomarkers, such as serum sST2 (<74.87 pg/mL) [15] and FC, according to the cut-off obtained from our patient cohort.

### Mucosal ST2 detection by immunofluorescence

Intestinal mucosa biopsies were reviewed by a pathologist experienced in IBD who was blinded to any clinical information about the patients. Mucosal tissue sections comprised the most inflamed segment according to chronic inflammatory infiltrate.

Biopsies were obtained at baseline and at 6 months or at baseline and 14 weeks in patients treated with conventional treatment and those rescued with biological therapy, respectively. Total ST2 content was detected in 2 % PFA-fixed, paraffin-embedded UC patient biopsies cut into 4-μm sections and subjected to immunofluorescence analysis. Non-specific binding was blocked and samples were incubated with a mouse monoclonal antibody against human ST2 (MAB523, R&D Systems) followed by an Alexa 546-tagged secondary goat antibody against mouse IgG (Invitrogen/Life Technologies, Carlsbad, CA, USA) to detect ST2. Cell nuclei were stained using Hoechst 33342 (Thermo/Life Technologies, Carlsbad, CA, USA). Images were captured using an Olympus confocal laser scanning biological microscope FV10i (Olympus America Inc., Melville, NY, USA) and processed using ImageJ (NIH, Bethesda, MD USA). Negative controls were prepared under conditions identical to those described above using an isotype control.

### Statistical analyses

Data were analyzed using GraphPad Prism5 (La Jolla, CA USA). Results of parametric numerical data are given as mean ± standard deviation (SD) and, in non-parametric distributions, as median and interquartile range (IQR). Serial assessments of change from baseline in serum ST2 and FC levels were compared using the Wilcoxon signed rank test. Differences and significance during follow-up times were analyzed by multiple comparisons using the non-parametric Kruskal-Wallis test with Dunn’s multiple comparison post-test. Correlations between sST2 levels and total intestinal ST2, FC, and clinical and activity score were analyzed using Spearman’s rank correlation coefficient (r). For each test, differences were considered significant at *p* ≤ 0.05.

## Results

### Serum ST2 and clinical characteristics of patients

Table 1 summarizes the clinical characteristics of 26 patients included in the protocol, while Fig. 1a shows the flow-chart of the 1-year follow-up enrollment and sampling overview at each stage of the patient’s monitoring.

**Table 1:** Clinical and demographic data of ulcerative colitis patients

	Number
Number of patients	26
Number of patients dropped out	2
Female/male	16/10
Median [range] age at entry	34.5 [30.0–52.5]
Duration of UC, years (median, range)	4.0 [1.3–11.8]
Age at diagnosis, years (median, range)	32.0 [22.5–44.0]
Location	
Proctitis	6
Left-sided	5
Extensive	15
Clinical disease activity	
Mild/ Moderate/ Severe	0/14/12
Endoscopic severity	
Moderate (score 2) / Severe (score 3)	22/4
Medication used	
No medication	3
5-ASA	11
Corticosteroids and 5-ASA	3
Corticosteroids, 5-asa and azathioprine	5
Corticosteroids and azathioprine	1
5-ASA and azathioprine	3
Response to therapy	21
Reactivation (after/before 6 months)	6 (1/5)
Rescued by anti-TNF**-**α	6

**Fig. 2:**
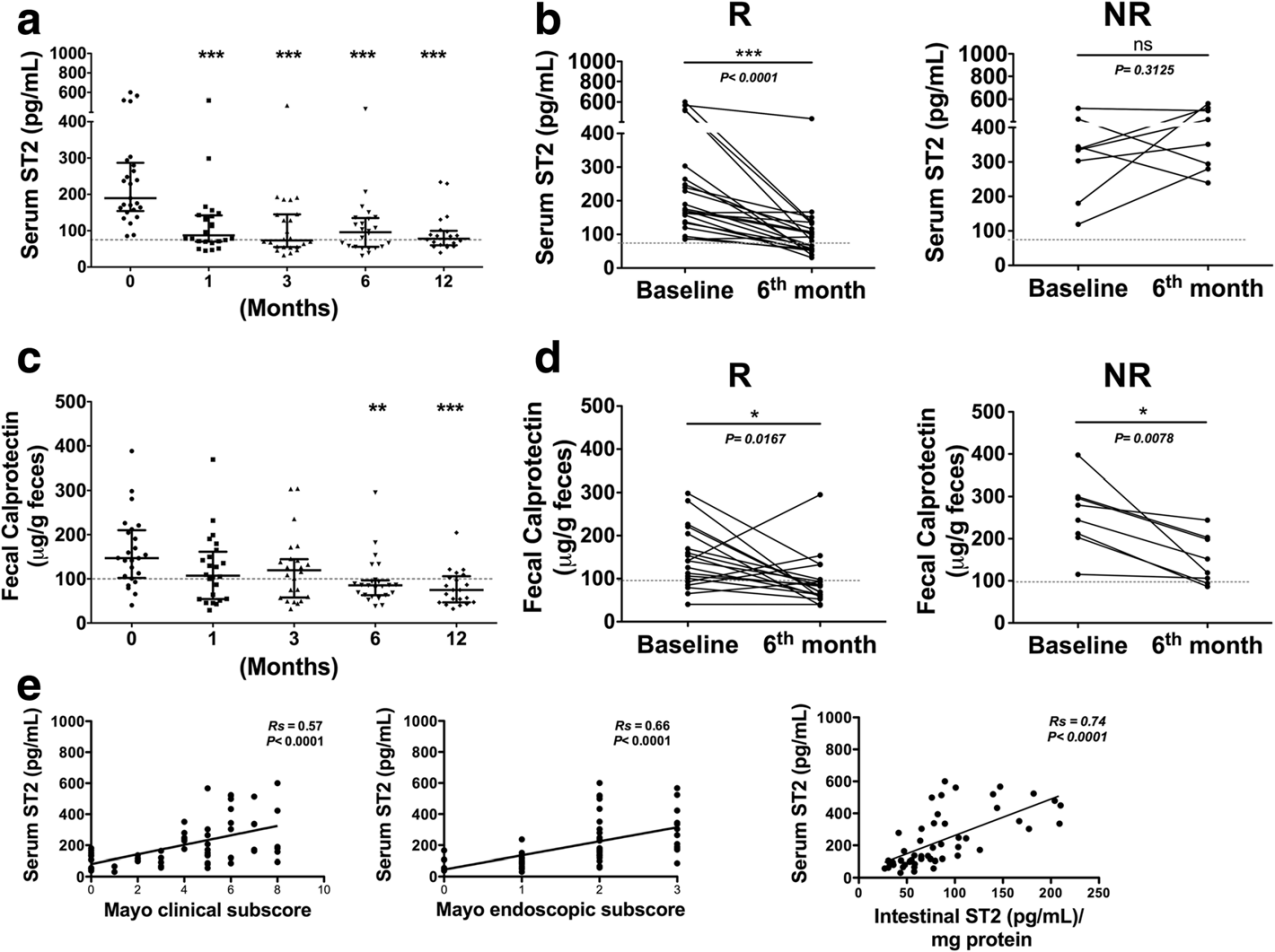
Distribution of serum ST2 according to response to therapy. **a** Serum ST2 levels during the follow-up period were determined in each patient and are shown as one symbol in the scatter plot. Horizontal lines indicate medians and whiskers (interquartile ranges). Differences were assessed using Kruskal-Wallis test (*** *p* < 0.0001). **b** Serum ST2 levels at baseline and 6 months in responders (R) and non-responders (NR) in relation to therapy decreased only in responders. Differences were assessed using Wilcoxon signed rank test (*** *p* < 0.0001). **c** FC levels in the 1-year follow-up and (**d**) at baseline and 6 months in R and NR in relation to therapy decreased in both subgroups. Panel E shows the correlation between serum ST2 levels and Mayo clinical subscore, Mayo endoscopic subscore, and total ST2 intestinal mucosa content, with a trend line for each correlation. Discontinuous line indicates cut-off value. Rs: Spearman’s rank correlation coefficient

Baseline ileo-colonoscopy data were available for 26 UC patients. During the study, 24 patients finished the protocol, six of whom showed disease reactivation, once in four patients after 6 months and twice in two patients before and after 6 months, for a total of eight reactivation episodes in six presumed non-responder patients. Overall, 18 patients responded to treatment and six patients did not (Fig. 1b). Baseline sST2 levels were 189.5 pg/mL [154.1–286.9]; at 1, 3, 6 and 12 months, with levels of 87.3 [70.1–142.2], 73.25 [55.1–145.1], 95.8 [55.7–134.9] and 78.0 [59.9–99.8] pg/mL, respectively (Fig. 2a). When patients were grouped according to treatment response, baseline levels were 173.5 [136.6–274.0], whereas sST2 levels at 6 months decreased to 86.5 [54.6–133.2] pg/mL in responders and increased in the eight reactivation episodes from 336.3 [211.0–403.2] to 385.3 [283.4–517.3] pg/mL in the six non-responders (Fig. 2b). No significant differences between therapy responder and non-responder patients were found in baseline sST2 values (*p* = 0.1057). Quantitation of FC at baseline, at 1, 3, 6 and 12 months revealed levels of 147.0 [102.1–210.2], 107.1 [54.2–161.3], 119.5 [57.7–144.5], 85.4 [63.0–96.7] and 74.7 [48.9–1076.0] μg/g feces, respectively (Fig. 2c). According to treatment, baseline FC levels of 141.4 [92.9–204.6] decreased to 82.8 [59.2–97.8] μg/g feces in responders and from 243.9 [175.1–297.9] to 152.0 [100.1–200.8] μg/g feces in non-responders at 6 months (Fig. 2d). sST2 levels in all follow-up times correlated with the Mayo clinical subscore (Rs = 0.57, *p* < 0.0001); at baseline and 6 months, levels correlated with Mayo endoscopic subscores (Rs = 0.66, *p* < 0.0001) and total ST2 content in mucosa (Rs = 0.74, *p* < 0.0001) (Fig. 2e). Patients in biological remission showed normalization of the inflammation biomarker sST2, with values below the cut-off of 74.87 pg/mL [15]. Considering this cut-off value of sST2, the specificity, sensitivity, PPV and NPV were 0.44, 0.95, 0.62, and 0.90, respectively.

When patients were grouped according to treatment response, IL-33 baseline levels were 199.8 [51.92–372.20], decreasing at 6 months to 141.2 [44.00–352.90] pg/mL in responders, and decreasing from 629.0 [49.56–716.8] to 217.9 [50.39–647.5] pg/mL in the reactivation episodes, both without significant differences (*p* > 0.05) (Additional file 1: Figure S1).

### Relationship of ST2 to the FC biomarker

**Fig. 3:**
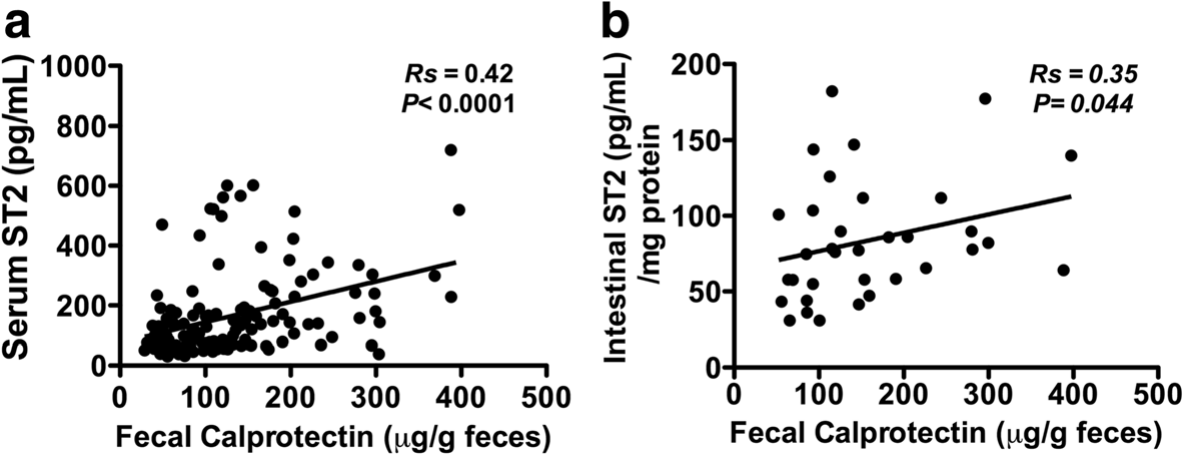
Correlation of ST2 and fecal calprotectin levels in patients. Serum ST2 and FC levels were determined at baseline, 1, 3, 6 and 12 months of follow-up. Total ST2 intestinal was measure at baseline and 6 months. Correlation between serum (**a**) and total intestinal ST2 (**b**) according to FC levels were assessed. Each symbol in the scatter plot represents the measurement in individual patients, with a trend line for this association. Rs: Spearman’s rank correlation coefficient (*p* < 0.05)

Analysis showed that sST2-circulating levels directly correlated with those of FC (Rs = 0.42, *p* < 0.0001) in all patients (Fig. 3a). Intestinal total ST2 levels in mucosa also correlated with those of FC, although the association was less significant statistically (Rs = 0.35, *p* = 0.044) (Fig. 3b).

### sST2 response to biological therapy

**Fig. 4:**
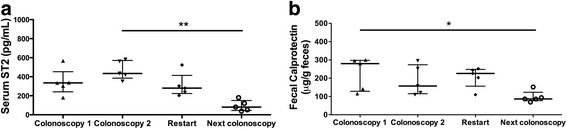
Distribution of serum ST2 and fecal calprotectin in patients rescued with biological therapy. Serum sST2 (**a**) and fecal calprotectin (**b**) levels are shown for patients non-responsive to conventional therapy or with reactivation but rescued with biological therapy. Each symbol in the scatter plot represents the measurement in individual patients; horizontal lines indicate medians and whiskers (the interquartile ranges). Differences were assessed using Kruskal-Wallis test with Dunn’s multiple comparison post-test (*p < 0.05 and **p < 0.01)

In our cohort, six patients were rescued with biological therapy. Immediately after infliximab infusions, sST2 levels were markedly increased, but protein content during the induction period gradually declined between weeks 3 and 24 of monitoring, reaching levels similar to those in patients responding to treatment (*p* < 0.01, Fig. 4a). A similar trend was observed for FC levels, indicating that fecal biomarker content also decreases gradually as a function of type and response to therapy (*p* < 0.05; Fig. 4b).

### Intestinal total ST2 immunoreactivity

**Fig. 5:**
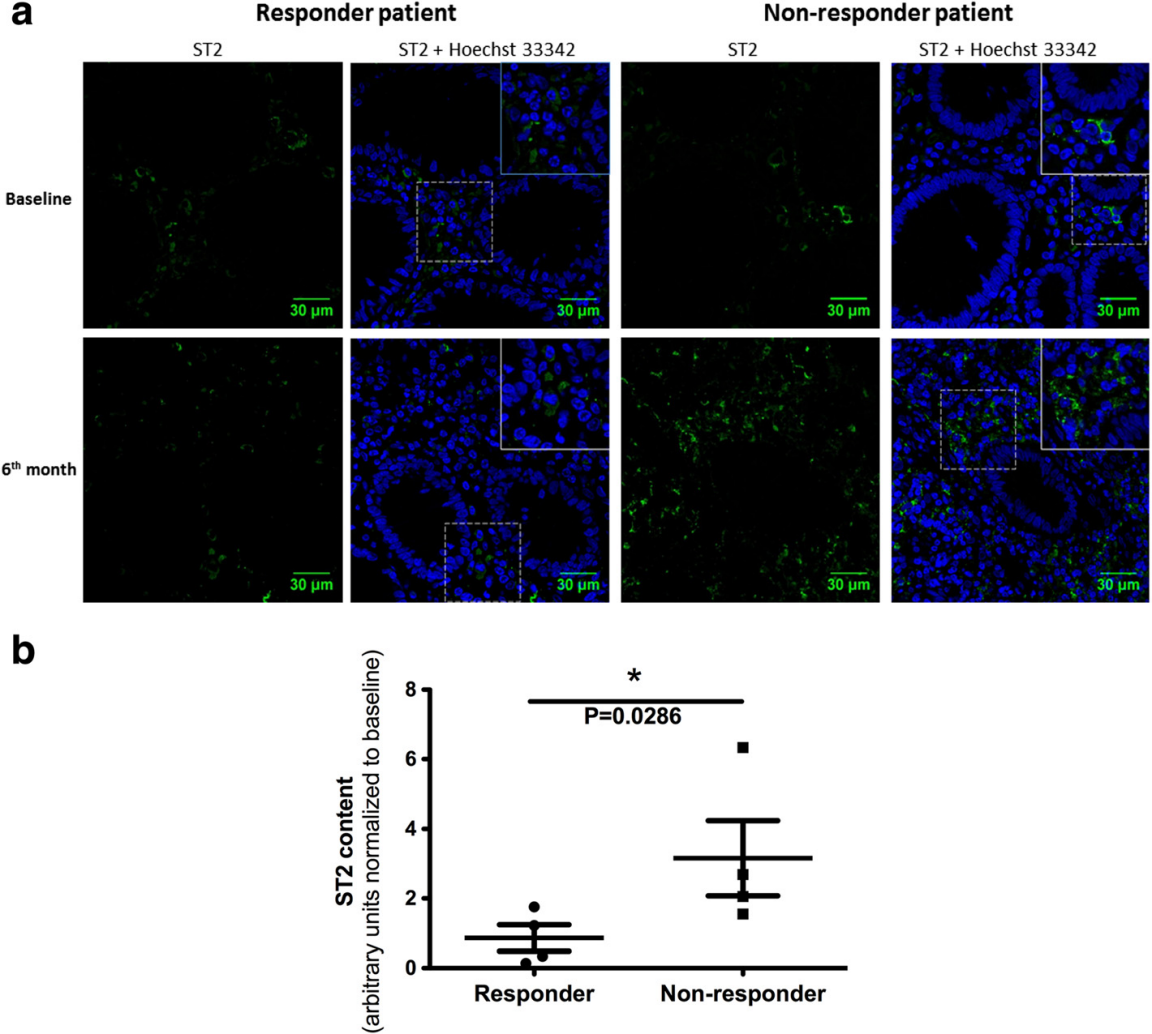
Immunolocalization of ST2 in colonic tissue from responder and non-responder patients during baseline and 6-month follow-up. **a** Total ST2 immunoreactivity was restricted to the cellular infiltrate in the lamina propria at 6 months in responder patients (right), while in patients showing reactivation, total ST2 was increased in inflamed mucosa, also confined to the cellular infiltrate (left); baseline examination revealed extensive immune cell infiltration of the intestinal mucosa and damaged tissue with loss of architecture. **b** Total ST2 immunoreactivity at 6 months expressed as arbitrary units (A.U.) and normalized to baseline levels in responders and non-responders (*n* = 4 in each group) was higher in the non-responders. Hoechst 33342/ ST2 (blue/green) (60X)

Because intestinal ST2 levels were highly correlated with sST2 concentrations (Fig. 2e), we tested whether the total mucosal ST2 content, as the source of ST2 in the periphery, might be related to healing and deep remission in responder patients. Mucosal ST2 immunoreactivity detected by immunofluorescence may allow identification of membrane-anchored ST2 and the soluble variant restricted to the intracellular compartment. In responder patients, total ST2 immunoreactivity was maintained in the cellular infiltrate of the lamina propria at 6 months of follow-up, whereas patients showing reactivation revealed increased total ST2 in inflamed mucosa but also confined to the cellular infiltrate (Fig. 5a,b). No immunoreactivity was detected in samples incubated with isotype antibody. Epithelial ST2 expression was absent in all responder and non-responder patients analyzed.

## Discussion

In this prospective study of UC patients with active disease, we consecutively measured sST2 levels during a 1-year follow-up and compared those with FC, the canonically defined biomarker of activity in IBD patients [23–25]. Our results demonstrate for first time that sST2 is an easily detectable marker of UC evolution and effectiveness of therapy.

Several studies reporting the association of the IL-33/ST2 signalling pathway with IBD pathogenesis in patients and animal models have attempted to address the actual contribution of each component of the pathway [26, 27]. In this context, the sST2 variant, acting as a decoy receptor for IL-33, is increased in UC [10, 15]. We proposed ST2 as a potential activity biomarker, with a cut-off value of 74.87 pg/mL, that might allow distinction between active and inactive disease with 83 % sensitivity and specificity [15]. At this cut-off value, we found a lower specificity (44 vs 83 %), suggestive of a poorer discrimination of the group of inactive patients or those with sST2 levels <74.87 pg/mL. The higher sST2 levels at baseline and from unresponsive patients/reactivations (endoscopic score ≥ 2) in the vast majority of all detections might explain the lower specificity.

sST2 has also been considered a biomarker of diseases that affect different tissues undergoing deep inflammatory reactions and necrosis; a cut-off of 150 pg/mL and 5.8 ng/mL was determined to predict sudden cardiac death in patients with chronic heart failure and left ventricular systolic dysfunction, as well as in patients with rheumatoid arthritis, respectively [28, 29].

In our follow-up study, patients in biological remission showed normalization of sST2, with values close to the cut-off previously determined [15], suggesting that low sST2 levels predict improvement in the inflammatory state. Moreover, we find that increased sST2 levels in UC patients with active disease directly correlate with higher expression of total ST2 in intestinal mucosa, including sST2 and ST2L, consistent with previous observations [15] and suggesting that the increased total ST2 immunoreactivity in non-responder patients is mostly at the expense of newly produced sST2 by cells infiltrating the mucosa. In responder patients, we found no conclusive evidence of a decrease in total ST2 immunoreactivity, possibly reflecting the inability of the technique used to distinguish between the two protein isoforms and its limitation in assessing protein quantity. Moreover, in responder patients, ST2+ cells infiltrating the mucosa might be less effective than those present in non-responders, although additional cell markers are needed to support this hypothesis. The components of this inflammatory microenvironment, including TNF-α, IL-33 and IL-1β produced by infiltrating and resident cells (neutrophils, mast cells and fibroblasts) have been shown to upregulate sST2 expression in vitro [30], although the current lack of a differential tool to detect ST2 variants limits the interpretation of tissue immunodetection of total ST2. On the other hand, studies of intestinal inflammation in murine models have reported that IL-33 itself can play a beneficial or harmful role in IBD depending on the induction of mucosal damage (acute or chronic) or the colitogenic used, arguing against a precise role of the IL-33/ST2 axis. Indeed, IL-33 serum levels did not correlate with the activity score in UC patients [15] and in the present cohort, we found no association between IL33 serum levels with therapy response.

The difficulty in evaluating disease activity in UC patients using clinical, endoscopic and histological approaches has led to greater interest in identifying new biochemical markers that directly reflect intestinal inflammation. Recently, the usefulness of FC as a biomarker has been explained by the fact that inflammation causes neutrophil activation, resulting in a proportional release-to-damage ratio. Yamamoto et al. [31] showed that consecutive measurements of FC in UC patients with proctitis were able to monitor relapse during maintenance therapy with mesalazine suppositories. A cut-off value of 55 μg/g was used to assign those patients who maintained clinical remission, although the optimal cut-off for defining activity in IBD remains controversial [32, 33]. Indeed, FC cut-off values ranging from 50 to 250 μg/g have been used [23, 34] that differ depending on the test used [35, 36]. In our study, we used quantitative ELISA and defined a cut-off of 99.26 μg/g with an AUC of 0.87 to distinguish individuals with an inactive endoscopic score (0–1) from those with activity indices (2–3) [37]. The cut-off value obtained for our cohort is similar to that previously described [38].

In 7 of 8 reactivations in non-responders from our cohort, FC levels showed a decrease despite detectable endoscopic inflammation, although FC values were above the cut-off defined in the current analysis. Unexpectedly, FC did not correlate with the endoscopic score, possibly related to the treatment received or presence of liquid stool [39]. Moreover, an inverse association between FC and neutrophil infiltration has been reported in patients under remission, with high protein levels in stool [40]. Finally, daily fluctuations of up to 40 % have been described for FC determined in four independent stool samples from active patients [41], suggesting that a single FC detection in active patients is not sufficient for a therapeutic decision.

In contrast, the increased inflammatory state of the mucosa in 5 of 8 reactivations of non-responders simultaneous with augmented sST2 levels indicates that this protein can predict lack of response to the therapy used. The direct association between endoscopic score and sST2 might reflect an inflammatory process related to mucosal damage, while the production and secretion of FC requires massive infiltration of neutrophils into the lamina propria [42, 43]. While no test can replace colonoscopy with biopsy to determine the inflammatory condition of the intestinal mucosa, the ability of sST2 levels to predict inflammatory status identifies the need to incorporate analysis of sST2 in selecting patients who require control for reactivation through colonoscopy.

sST2 levels have been also identified as a biomarker in heart failure, efficiently assessing cardiac remodelling and fibrosis; sST2 has been shown to efficiently monitor the effectiveness of an optimized treatment in chronic heart failure patients [44], with increased levels that may be a good predictor of cardiac decompensation as well as worsening renal function.

To date, there are few reports on the behavior of sST2 according to the medication used in IBD patients. We found some association of sST2 with the use of systemic steroids [15], and others have reported the effect of infliximab on this molecule [10]. However, no study has demonstrated how ST2 levels fluctuate in serial and continuous follow-up in relation to endoscopic index.

Based on our previous finding [15], we recruited patients who at baseline had a moderate or severe endoscopic index (≥2) in any analyzed section of the colon. During the follow-up, serial ST2 measurements decreased in those patients with a reduced endoscopic index at 6 months, indicating a positive response to therapy. In those patients, FC levels were also significantly decreased in direct correlation to sST2 levels. Further association studies between sST2 and FC in a larger cohort of UC patients are needed, as well as such analyses in CD and in IBD pediatric patients. Correlation studies between sST2, histologic scores and disease risk might also provide additional information about the strength of this biomarker as has been demonstrated with FC [45].

Our analyses of a one-bounded cohort of UC patients during a 1-year follow-up revealed a direct correlation between sST2 levels and inflammatory activity, strongly suggesting that sST2 is a reliable biomarker of active UC. It will be interesting to examine the predictive role of sST2 for reactivation risk in a cohort of inactive patients or undergoing clinical and endoscopic remission (Mayo subscore 0) and identifying serum levels of this biomarker in a follow-up every 4 months, a protocol in which FC was shown to predict disease recurrence [31]. Finally, it will be interesting to monitor both the behavior of sST2 in a cohort of severe UC patients and the relevance of elevated sST2 content in predicting colectomy risk in a 1-year follow-up, as shown for FC [46].

## Conclusions

The accuracy of sST2 in endoscopic detection of UC strongly suggests its usefulness in monitoring relapse and outcome, as well as in identifying patients likely to benefit from a particular treatment.

## Additional file

Additional file 1: Figure S1. Distribution of serum IL33 according to response to therapy. Serum IL33 levels at baseline and 6 months in responders (R) and non-responders (NR) in relation to therapy without significant differences (*p* > 0.05). Differences were assessed using Wilcoxon signed rank test. (TIFF 25551 kb)

## Abbreviations

5-ASA: 5-aminosalicylate derivatives
CD: Crohn’s disease
ELISA: Enzyme-linked immunosorbent assay
FC: Fecal calprotectin
IBD: Inflammatory bowel diseases
sST2: Soluble ST2
UC: Ulcerative colitis
