# Supplementary figures and images for: Soluble ST2 is a sensitive clinical marker of ulcerative colitis evolution
Source: BMC Gastroenterol. 2016 Aug 26;16(1):103. doi: 10.1186/s12876-016-0520-6 (PMC5002140; doi:10.1186/s12876-016-0520-6)

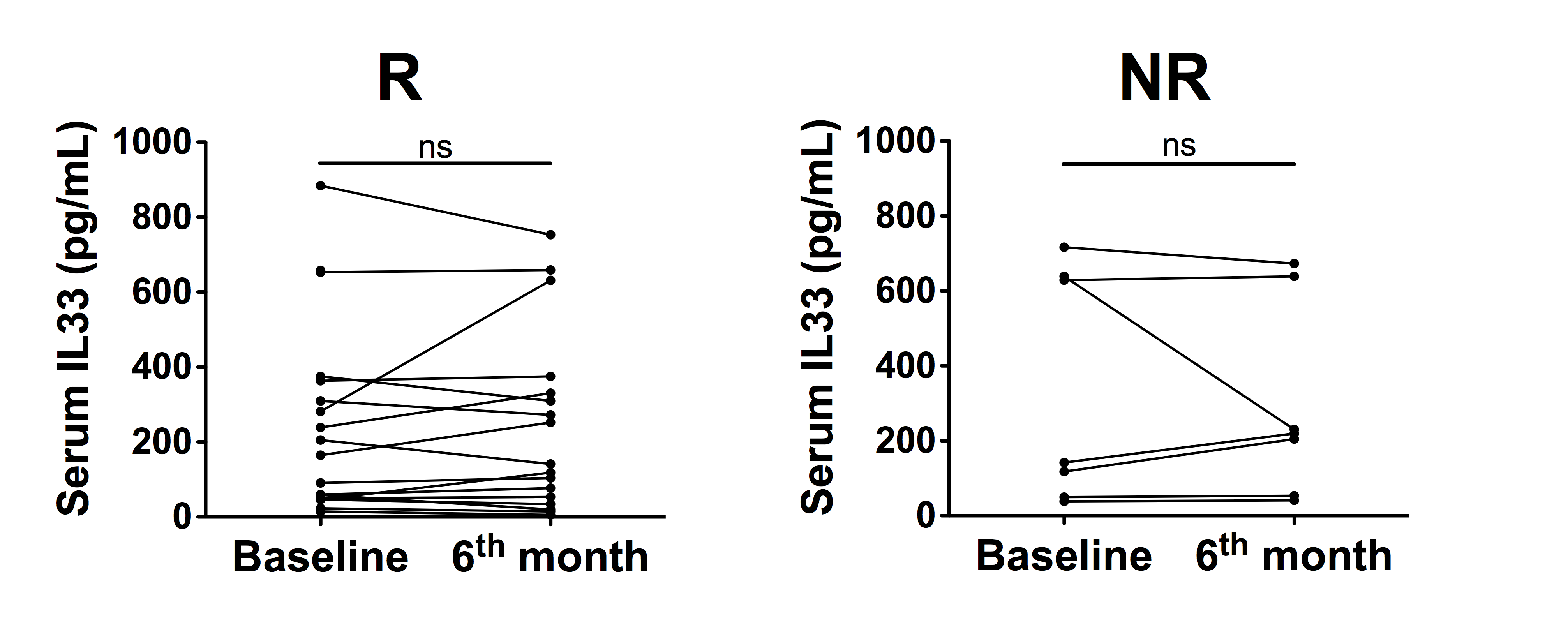

Supplement: Additional file 1: Figure S1. — Distribution of serum IL33 according to response to therapy. Serum IL33 levels at baseline and 6 months in responders (R) and non-responders (NR) in relation to therapy without significant differences (p > 0.05). Differences were assessed using Wilcoxon signed rank test. (TIFF 25551 kb) [file 12876_2016_520_MOESM1_ESM.tiff]
